# Expression significance and potential mechanism of hypoxia‐inducible factor 1 alpha in patients with myelodysplastic syndromes

**DOI:** 10.1002/cam4.2447

**Published:** 2019-08-14

**Authors:** Hai‐wei Liang, Bin Luo, Li‐hua Du, Rong‐quan He, Gang Chen, Zhi‐gang Peng, Jie Ma

**Affiliations:** ^1^ Department of Pathology The First Affiliated Hospital of Guangxi Medical University Nanning People's Republic of China; ^2^ Department of Medical Oncology The First Affiliated Hospital of Guangxi Medical University Nanning People's Republic of China

**Keywords:** bioinformatics, hypoxia‐inducible factor 1 alpha, immunohistochemistry, myelodysplastic syndromes, prognosis

## Abstract

**Objective:**

To investigate the expression level and potential mechanism of hypoxia‐inducible factor 1 alpha (HIF‐1α) in patients with myelodysplastic syndromes (MDS).

**Methods:**

Immunohistochemistry (IHC) techniques were used to examine the protein expression of HIF‐1α in paraffin‐embedded myeloid tissues from 82 patients with MDS and 33 controls (patients with lymphoma that is not invading myeloid tissues). In addition, the associations between the protein expression of HIF‐1α and clinical parameters were examined. To further investigate the significance of HIF‐1α expression in MDS patients, the researchers not only extracted the data about HIF‐1α expression from MDS‐related microarrays but also analyzed the correlation between the level of HIF‐1α expression and MDS. The microRNA (miRNA) targeting HIF‐1α was predicted and verified with a dual luciferase experiment.

**Results:**

Immunohistochemistry revealed that the positive expression rate of HIF‐1α in the bone marrow of patients with MDS was 90.24%. This rate was remarkably higher than that of the controls (72.73%) and was statistically significant (*P* < .05), which indicated that HIF‐1α was upregulated in the myeloid tissues of MDS patients. For the GSE2779, GSE18366, GSE41130, and GSE61853 microarrays, the average expression of HIF‐1α in MDS patients was higher than in the controls. Particularly for the GSE18366 microarray, HIF‐1α expression was considerably higher in MDS patients than in the controls (*P* < .05). It was predicted that miR‐93‐5p had a site for binding with HIF‐1α, and a dual luciferase experiment confirmed that miR‐93‐5p could bind with HIF‐1α.

**Conclusion:**

The upregulated expression of HIF‐1α was examined in the myeloid tissues of MDS patients. The presence of HIF‐1α (+) suggested an unsatisfactory prognosis for patients, which could assist in the diagnosis of MDS. In addition, miR‐93‐5p could bind to HIF‐1α by targeting, showing its potential to be the target of HIF‐1α in MDS.

## Graphical abstract

The up‐regulated expression of hypoxia‐inducible factor 1 alpha was examined in the myeloid tissues of myelodysplastic syndromes (MDS) patients. In terms of immunohistochemistry staining intensity, the MDS cohort (Figures B&D) displayed more intense staining than the controls (Figures A&C).

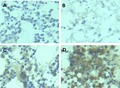

## 1 INTRODUCTION

Myelodysplastic syndromes (MDS) are defined as a category of clonal myeloid disorders that are prone to lead the development of acute myelocytic leukemia (AML).^1^, ^2^, ^3^, ^4^ Currently, researchers are not very knowledgeable about the complex pathogenesis of MDS. However, angiogenesis has been regarded as a vital pathophysiological process in solid tumors for tumor growth, proliferation, and metastasis.^5^, ^6^, ^7^, ^8^, ^9^, ^10^ In addition, recent studies showed that angiogenesis and angiogenesis factors play a vital role in the onset and development of MDS and AML.^11^, ^12^, ^13^, ^14^ Various types of angiogenesis factors were observed in the bone marrow of patients with AML or MDS, including vascular endothelial growth factor (VEGF), fibroblast growth factor (bFGF), angiogenin, angiogenin‐1, platelet‐derived growth factor (PDGF), hepatocyte growth factor (HGF), epidermal growth factor (EGF), tumor necrosis factor alpha (TNF‐α), transforming growth factor alpha (TGF‐α), and transforming growth factor beta (TGF‐β). Most of these factors were secreted by hematopoietic cells in tumors, promoting the growth and proliferation of leukemia cells via an autocrine mechanism.^14^, ^15^, ^16^, ^17^, ^18^, ^19^

Hypoxia‐inducible factor 1 alpha (HIF‐1α), a subunit that maintains the activity of hypoxia‐inducible factor 1 (HIF‐1), is the key regulatory factor inducing the expression of genes related to cell survival and adaptation in hypoxia conditions.^20^, ^21^, ^22^ HIF‐1 is considered to be important for regulating the oxygen balance in cells and mediating hypoxic reactions; therefore, it is correlated with the onset and development of tumors. A multitude of HIF‐1α‐mediated biological effects could promote tumor development, such as metabolic transition to glycolysis, stimulation of tumor angiogenesis, and alteration in the expression of tumor‐inhibiting genes.^23^, ^24^, ^25^, ^26^ In addition, the formation of new vessels greatly affects the restoration of damaged tissues, which requires a supply of oxygen and nutrients, especially in tumor tissues.^27^, ^28^, ^29^ In breast carcinoma that arises under hypoxic conditions, HIF‐1 could mediate the protein‐coding genes for transcriptional activation.^30^ These proteins are indispensable for tumor progression and the formation of new vessels.^31^ Recently, a large number of studies revealed that HIF‐1α was an essential transcriptional factor involved in angiogenesis, which was overexpressed in some types of tumors and accelerated tumors’ initiation and progression^32^, ^33^, ^34^ by, for example, stimulating relevant genes, like VEGF.^35^, ^36^, ^37^, ^38^

Scholars have defined microRNA (miRNA) as a small noncoding single‐stranded RNA that can bind to target genes, inhibiting the protein translation of the target genes or degrading the mRNA and thus regulating the expression of downstream target genes and their biological functions.^39^, ^40^, ^41^, ^42^, ^43^ Studies have shown that HIF‐1α can be the target gene of various miRNAs. The interactions between theses miRNAs and HIF‐1α can explain some important events related to tumorigenesis, such as angiogenesis, metabolism, apoptosis, cell cycle regulation, proliferation, metastasis, and resistance to anticancer therapy.

To determine the significance of HIF‐1α expression in MDS patients, the expression level of HIF‐1α in MDS was examined with immunohistochemistry (IHC), the Gene Expression Omnibus (GEO) database, and the ArrayExpress database. The miRWalk2.0, an online prediction website, claimed that miR‐93‐5p had complementary sites for binding with HIF‐1α. Therefore, a dual luciferase experiment was carried out to see whether miR‐93‐5 could target HIF‐1α to perform its biological functions in MDS patients.

## 2 MATERIALS AND METHODS

### 2.1 Cell culture

The researchers purchased 293T cells from the Shanghai Institutes for Biological Sciences and then cultured the cells with DMEM medium (Corning) that contained 10% fetal bovine serum (Ausbian), 100 U/mL penicillin, and 100 U/mL streptomycin. The cells were cultivated in a CO_2_ incubator with 5% CO_2_, 95% humidity, and a temperature of 37°C. The medium was changed daily, and the cells were extracted at the logarithmic phase for future experiments.

### 2.2 Cases and samples

Paraffin‐embedded myeloid tissues from 82 patients with MDS and 12 patients with AML were obtained from the First Hospital Affiliated to Guangxi Medical University between October 2012 and December 2016. According to the expert consensus on diagnosis and treatment of myelodysplastic syndrome,^44^ all the MDS patients were diagnosed based on the 2008 World Health Organization's criteria for MDS. Patients, examined with a fluorescence in situ hybridization (FISH) test or karyotype analysis, were categorized into different groups based on the International Prognostic Scoring System (IPSS) for MDS.^45^ For the controls, the researchers selected the paraffin‐embedded tissues of 33 patients with lymphoma that was not invading the bone marrow (Table 1). The present study was approved by the Research Ethics Committee of the First Affiliated Hospital of Guangxi Medical University (Nanning, China), and written informed consent was obtained from all patients.

**Table 1:** The relationships between HIF‐1α expression in the myeloid tissues of MDS patients and clinicopathologic parameters

	Percentage of cases				
	Cases	HIF‐1α (−) HIF‐1α (+)		*χ* ^2^	*P* value
Tissue 1					
MDS	82	8 (9.76)	74 (90.24)	4.43	<.05*
Controls	33	9 (27.27)	24 (72.73)		
Conversion to AML					
Yes	12	2 (16.67)	10 (83.33)	0.05	.82
No	82	8 (9.76)	74 (90.24)		
Tissue 2					
Conversion to AML	12	2 (16.67)	10 (83.33)	0.12	.73
Controls	33	9 (27.27)	24 (72.73)		
Age					
≥51	43	3 (6.98)	40 (93.02)	0.27	.60
<51	39	5 (12.82)	34 (87.18)		
Sex					
Male	49	7 (14.29)	42 (85.71)	1.7	.19
Female	33	1 (3.03)	32 (96.97)		
WHO classification 1					
RA/RARS/5q‐	10	1 (10.00)	9 (90.00)	—	.61
RAEB1	21	1 (4.76)	20 (95.24)		
RAEB2	19	1 (5.26)	18 (94.74)		
RCMD	32	5 (15.63)	27 (84.38)		
WHO classification 1					
RAEB1/RAEB2	40	2 (5.00)	38 (95.00)	1.09	.30
RA/RARS/RCMD/5q‐	42	6 (14.29)	36 (85.71)		
IPSS					
High risk	10	1 (10.00)	9 (90.00)	—	1.00
Low risk/moderate risk‐1/moderate risk‐2	61	5 (8.20)	56 (91.80)		
Prognosis of karyotype					
Unsatisfactory prognosis	26	4 (15.38)	22 (84.62)	1.26	.26
Good/average prognosis	44	2 (4.55)	42 (95.45)		
Initial cells					
≥5%	38	2 (5.26)	36 (94.74)	0.63	.43
<5%	38	5 (13.16)	33 (86.84)		

Abbreviations: AML, acute myeloid leukemia; HIF‐1α, hypoxia‐inducible factor 1 alpha; IPSS, International Prognostic Scoring System; MDS, myelodysplastic syndrome.

* *P* < .05

### 2.3 Bioinformatics prediction and the dual luciferase experiment

For the purpose of exploring the biological role of HIF‐1α in MDS, the miRNAs targeting HIF‐1α were predicted using bioinformatics techniques and four databases (miRWalk, miRanda, RNA22, and Targetscan) on the miRWalk2.0 website.^46^ The mediums for wild‐type HIF‐1α 3'UTR, mutant‐type HIF‐1α 3'UTR, and overexpressed miR‐93‐5p were created separately. The gene orders of amplified wild‐type HIF‐1α 3'UTR, mutant‐type HIF‐1α 3'UTR, and overexpressed miR‐93‐5p were linked to GV272 and GV268 mediums (Shanghai Genechem Co., Ltd.) after digestion. Eventually, the colony containing the mediums for wild‐type HIF‐1α 3'UTR, mutant‐type HIF‐1α 3'UTR, and miR‐93‐5p was obtained. We chose 293T cells at the logarithmic growth phase for transfection and divided the samples for the experiment into four cohorts: the negative controls miRNA‐NC and 3'UTR‐NC (miR‐93‐5p no‐load plasmid and 3'UTR no‐load plasmid), miRNA (miR‐93‐5p plasmid vector), 3'UTR (HIF‐1A 3'UTR plasmid), and 3'UTR‐MU (HIF‐1α 3'UTR mutant plasmid). The dual‐luciferase reporter assay system (Promega) was applied for luciferase activity assay of the samples. Forty‐eight hours after transfection, the used medium was discarded. Phosphate‐buffered saline (PBS) was used to wash the cells twice, and then passive lysis buffer was added. After cell lysis, the researchers used the dual‐luciferase reporter assay system to perform a luciferase activity assay for each cohort.

### 2.4 Immunohistochemistry

The paraffin‐embedded tissues were serially sectioned into 4‐μm parts and warmed at 75°C for 30 minutes. Ethylenediamine tetra acetic acid retrieval solution (Maxim Biotechnology Development Co., Ltd.) was added to a pressure cooker and heated. The sections were soaked in 3% hydrogen peroxide for 15 minutes to block endogenous peroxidase. Next, the primary antibody was added. The sectioned tissues were treated with HIF‐1α and anti‐PDGF‐B antibody (Abcam) and then incubated at 37°C for 1.5 hours. Subsequently, the secondary antibody, SupervisionTM mouse/rabbit secondary antibody, was incubated at room temperature for 30 minutes. After washing with PBS, the sections underwent 3,3'‐diaminobenzidine staining (ZSGB‐BIO Corp), were examined under the microscope, and then were re‐dyed with hematoxylin. Finally, dehydration and pH‐neutral resin mounting were performed. The confirmed positive tissue sections, according to the expert consensus on diagnosis and treatment of myelodysplastic syndrome (2014), acted as positive controls, while sections with PBS as a substitute for the primary antibody were chosen as the negative controls.^47^

### 2.5 IHC results

The results of IHC staining of HIF‐1α were as follows^48^:

1. negative staining: −;
2. positive staining cells <1%: +;
3. positive staining cells 1%‐10% and/or cytoplasmic staining: ++;
4. positive staining cells 10%‐50% and/or moderate cytoplasmic staining: +++;
5. positive staining cells >50% and/or intensive cytoplasmic staining: ++++.

### 2.6 Collection and selection of microarrays and literature

The researchers retrieved microarrays related to MDS from the GEO and ArrayExpress databases with the searching strategies: (“dysmyelopoietic syndrome” OR “dysmyelopoietic syndromes” OR “myelodysplastic syndrome” OR “myelodysplastic syndromes” OR “hematopoetic myelodysplasia” OR “hematopoetic myelodysplasias” OR “MDS”). The inclusion standards were as follows: gene expression microarrays of the MDS patients and controls are involved; gene expression microarrays of MDS and AML are available; and data concerning the expression of HIF‐1α are contained in the microarray, including the mean and SD values. Therefore, the researchers eliminated microarrays that lacked controls, contained no data concerning the expression of HIF‐1α, or included only one case as well as those for which mean or SD values were unavailable.^49^

### 2.7 Meta‐analysis

For the acquired microarrays, the researchers extracted data concerning the expression of HIF‐1α, calculated the mean and SD values, and employed Stata 14.0 for meta‐analysis. If *P* < .05 and *I* ^2^ > 50%, it was determined that heterogeneity existed, and the random effects model was used. If *P* ≥ .05 and *I* ^2^ ≤ 50%, it was determined that homogeneity existed, and the fixed effects model was utilized. Subsequently, forest maps and funnel plots were used to illustrate the analysis. Stata 14.0 was employed to carry out the meta‐analyses of measuring the summary receiver operating characteristic (SROC), helping the researchers draw SROC curves.

### 2.8 Statistical analysis

SPSS 22.0 was used for statistical analysis, and SPSS 22.0 or GraphPad Prism 7.0 was applied for making graphs. The researchers also used Student's *t* test for comparing two cohorts of data. For comparisons within a cohort, the researchers used the chi‐squared test or exact probability test. Statistical significance was set at *P *< .05.

## 3 RESULTS

### 3.1 Expression and clinical significance of HIF‐1α in MDS myeloid tissue sections

**Figure 1:**
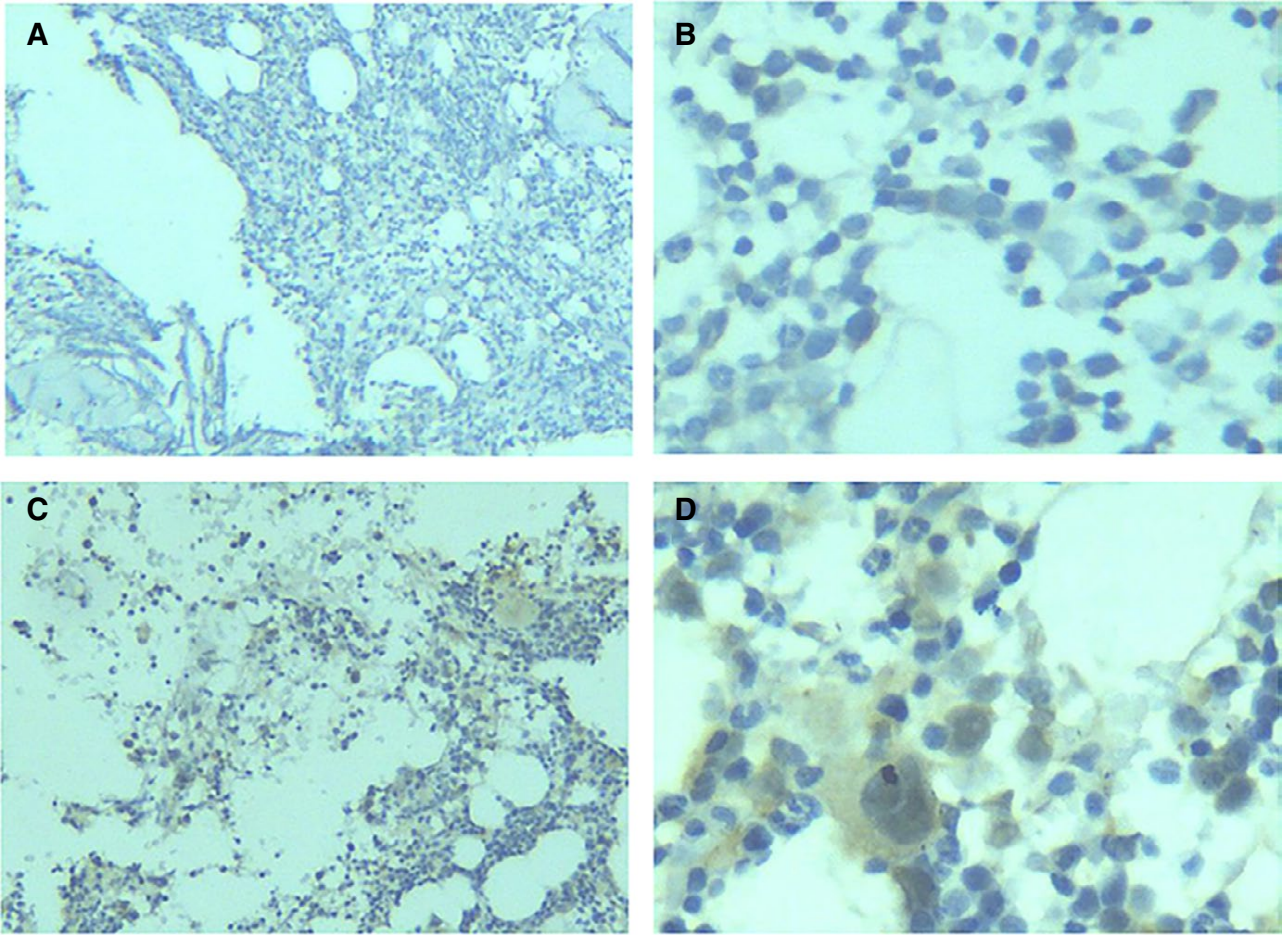
HIF‐1α expression in the myeloid tissues of the controls. (A), After the myeloid tissues of the controls underwent IHC staining, HIF‐1α was negatively expressed (100×). (B), After the myeloid tissues of the controls underwent IHC staining, HIF‐1α was negatively expressed (400×). (C), After the myeloid tissues of the controls underwent IHC staining, HIF‐1α was positively expressed (100×). (D), After the myeloid tissues of the controls underwent IHC staining, HIF‐1α was positively expressed (400×). HIF‐1α, hypoxia‐inducible factor 1 alpha; IHC, immunohistochemistry

**Figure 2:**
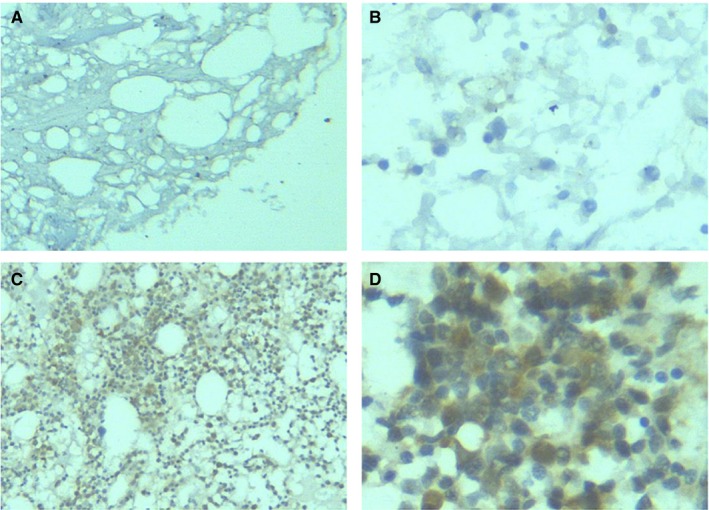
HIF‐1α expression in the myeloid tissues of MDS patients. (A), After the myeloid tissues of MDS patients underwent IHC staining, HIF‐1α was negatively expressed (100×). (B), After the myeloid tissues of MDS patients underwent IHC staining, HIF‐1α was negatively expressed (400×). (C), After the myeloid tissues of MDS patients underwent IHC staining, HIF‐1α was positively expressed (100×). (D), After the myeloid tissues of MDS patients underwent IHC staining, HIF‐1α was positively expressed (400×). HIF‐1α, hypoxia‐inducible factor 1 alpha; IHC, immunohistochemistry; MDS, myelodysplastic syndrome

**Figure 3:**
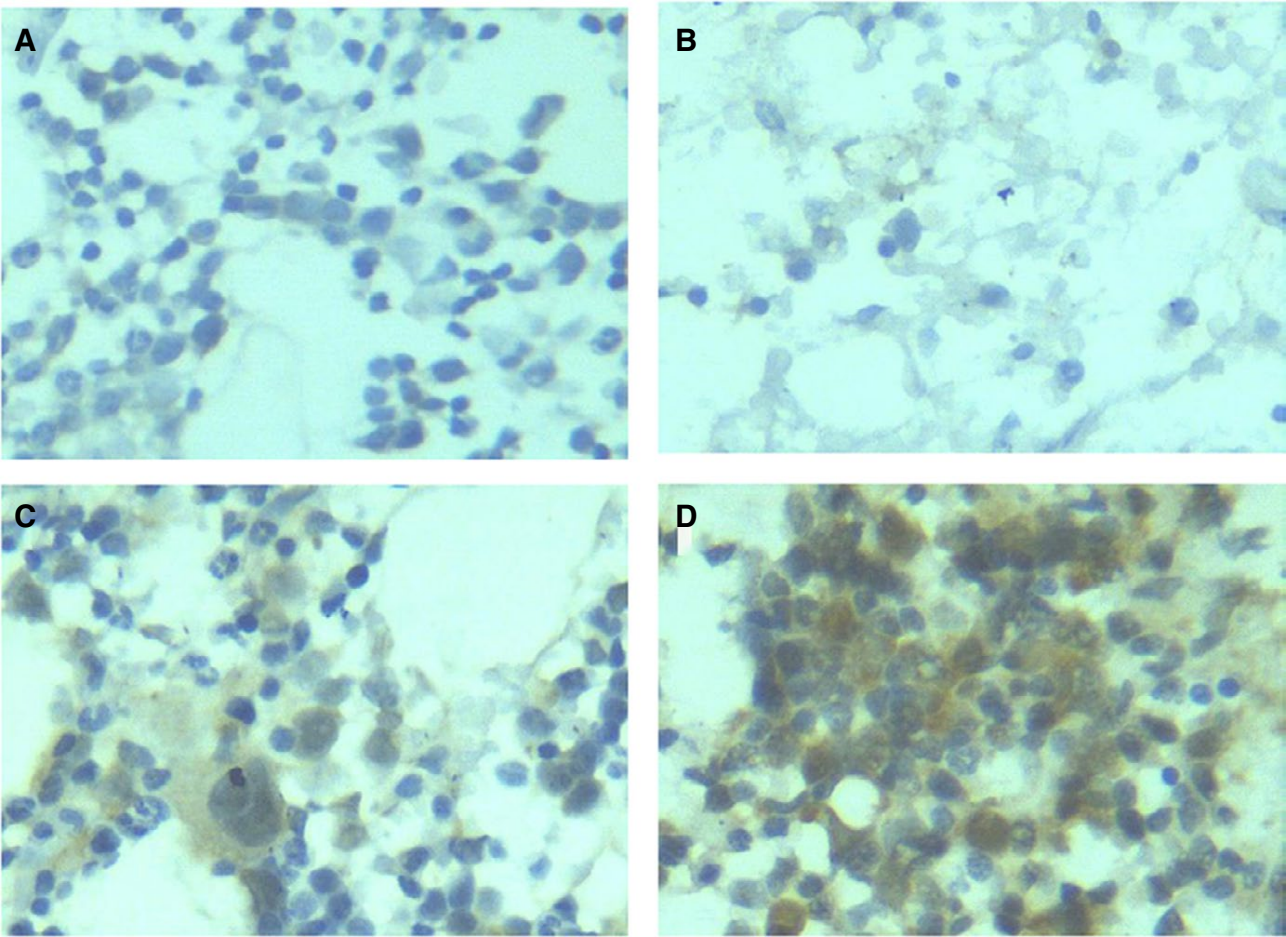
HIF‐1α expression in the myeloid tissues of MDS patients and controls. (A), After the myeloid tissues of the controls underwent IHC staining, HIF‐1α was negatively expressed. (400×). (B), After the myeloid tissues of MDS patients underwent IHC staining, HIF‐1α was negatively expressed (400×). (C), After the myeloid tissues of the controls underwent IHC staining, HIF‐1α was positively expressed (400×). (D), After the myeloid tissues of MDS patients underwent IHC staining, HIF‐1α was positively expressed (400×). HIF‐1α, hypoxia‐inducible factor 1 alpha; IHC, immunohistochemistry; MDS, myelodysplastic syndrome

In this study, IHC staining was conducted on myeloid tissues from 82 MDS patients and 33 controls, and HIF‐1α expression was evaluated in three types of tissues (Figures 1, 2, 3). It was discovered that HIF‐1α was expressed in the cell nuclei and cytoplasm of myeloid tissues. Of the 82 MDS cases, 74 showed positive HIF‐1α expression and 8 cases showed negative expression. Therefore, HIF‐1α (+) represented 90.24% of the cases. Of the 33 control cases, HIF‐1α was expressed positively in 24 cases and negatively in 9 cases. Therefore, HIF‐1α (+) was presented in 72.73% of the cases. In terms of staining intensity, the MDS cohort displayed more intense staining than the controls (Figure 3). Compared with the controls (72.73%), the percentage of positively expressed HIF‐1α (90.24%) was clearly higher in the MDS cohort. The chi‐squared test showed that HIF‐1α expression was statistically significant in the MDS group and controls (*P* < .05), indicating that increased HIF‐1α expression could be found in the myeloid tissues of MDS patients (Table 1).

**Figure 4:**
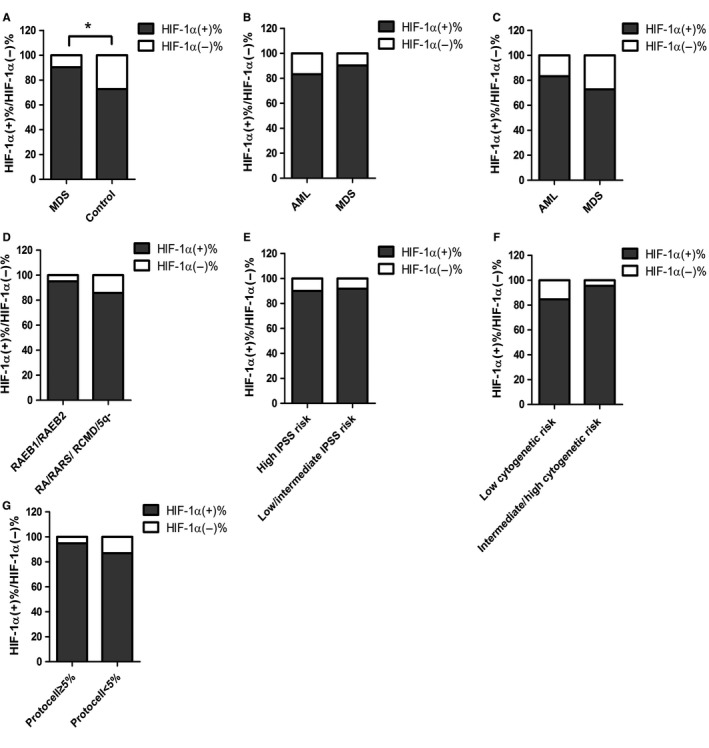
The relationships between HIF‐1α expression in the myeloid tissues of MDS patients and clinicopathologic parameters. HIF‐1α, hypoxia‐inducible factor 1 alpha; MDS, myelodysplastic syndrome. *P < .05

The expression level of HIF‐1α in the bone marrow tissues of MDS was not statistically significant in relation to clinicopathologic parameters, such as WHO classification, IPSS scores, karyotype prognosis, and the cell type (Table 1, Figure 4). In the RAEB1/RAEB2 cohort, the rate of positive HIF‐1α expression was 95.00%, much higher than that in RA/RARS/RCMD/5q‐ cohort (85.71%). In addition, the rate of positive HIF‐1α expression was higher for cases with an initial cell rate of ≥5% (94.74%) than in cases with an initial cell rate of <5% (86.84%). Furthermore, the rate of positive HIF‐1α expression was higher for items related to unsatisfactory prognosis (RAEB1/RAEB2, initial cell ≥5%). RAEB1/RAEB2 comprised 51.35% of the HIF‐1α (+) cohort, remarkably higher than in the HIF‐1α (−) cohort (25.00%). In addition, the average rate of the initial cell was 5.73% in the HIF‐1α (+) cohort and 3.34% in the HIF‐1α (−) cohort. Since RAEB1/RAEB2 tended to develop into AML, and an initial cell rate of more than 5% indicates poor prognosis, RAEB1/RAEB2 and an initial cell rate of ≥5% could be used as indicators of unsatisfactory prognosis. Thus, positively expressed HIF‐1α was associated with unsatisfactory prognosis in MDS patients.

**Table 2:** The relationships between HIF‐1α expression in the myeloid tissues of MDS patients and blood‐related clinicopathologic parameters

Blood‐related parameters	Median value (range)		T value	*P* value
	HIF‐1α (−)	HIF‐1α (+)		
Initial cells (%)	0.50 (0.00‐13.20)	5.00 (0.00‐18.50)	−1.16	.25
Leukocytes (×10^9^/L)	2.40 (1.52‐3.30)	3.06 (0.98‐10.53)	−3.57	<.001***
Hemoglobin (g/L)	64.00 (40.30‐108.00)	67.00 (26.30‐113.40)	0.18	.86
Platelets (×10^9^/L)	22.50 (2.00‐387.00)	73.30 (4.30‐477.80)	−0.85	.40
Neutrocytes (×10^9^/L)	1.20 (0.70‐2.10)	1.49 (0.08‐8.70)	−2.25	<.05*
Lactate dehydrogenase (μ/L)	174.00 (9.00‐732.00)	242.00 (105.00‐2777.00)	−0.24	.81
Microglobulin (μg/L)	3.55 (2.12‐6.54)	3.20 (1.42‐6.33)	1.19	.24

Abbreviations: HIF‐1α, hypoxia‐inducible factor 1 alpha; MDS, myelodysplastic syndrome.

* *P* < .05.

*** *P* < .001.

**Figure 5:**
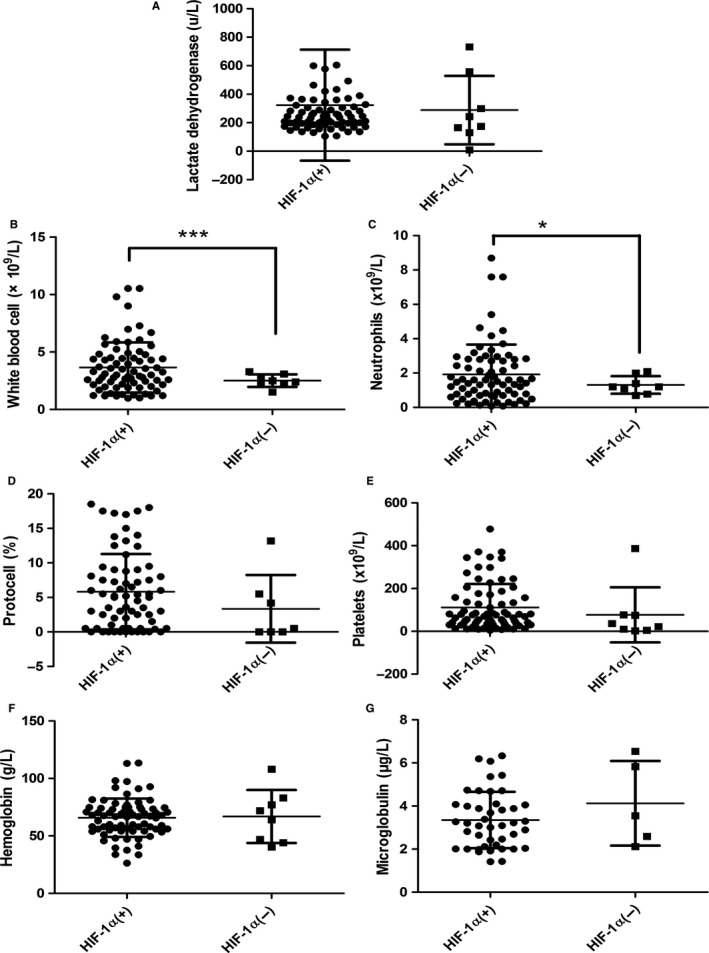
The relationships between HIF‐1α expression in the myeloid tissues of MDS patients and blood‐related clinicopathologic parameters. HIF‐1α, hypoxia‐inducible factor 1 alpha; MDS, myelodysplastic syndrome. *P < .05, ***P < .001

The researchers also investigated the correlations between blood‐relevant clinical parameters and HIF‐1α expression, finding that HIF‐1α expressed in the myeloid tissues of MDS patients was statistically significant in terms of the number of leukocytes (*P* < .001) and the number of neutrocytes (*P* < .05). Nevertheless, no statistical significance was found in terms of the initial cell, hemoglobin, platelets, lactate dehydrogenase, or microglobulin. Despite this, the mean value of lactate dehydrogenase was greater in cases of HIF‐1α (+) (314.54 µ/L) than in cases of HIF‐1α (−) (288.75 µ/L). This suggests that HIF‐1α is correlated with poor prognosis; the increased level of lactate dehydrogenase negatively affects the medical outcomes of hematologic malignancies (Table 2, Figure 5).

### 3.2 Expression and significance of HIF‐1α in MDS‐related microarrays

**Figure 6:**
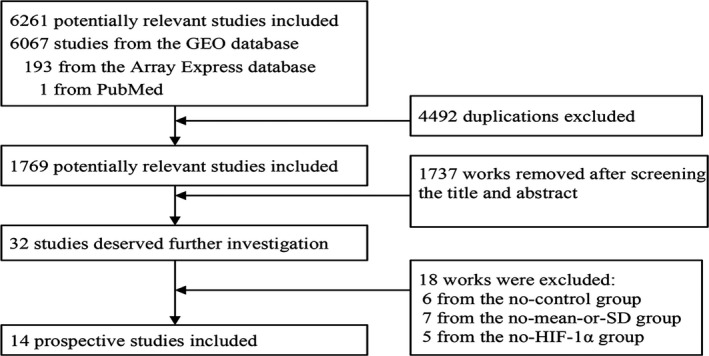
Flow chart illustrating the selection of MDS‐related microarrays from the literature. GEO, Gene Expression Omnibus; HIF‐1α, hypoxia‐inducible factor 1 alpha; MDS, myelodysplastic syndrome; SD, standard deviation

A total of 14 microarrays were included in our research (Table 3). These microarrays were searched for and selected from the GEO and ArrayExpress databases (Figure 6). In all 14 microarrays, which contained data about the controls, HIF‐1α was more highly expressed in GSE2779, GSE18366 (*P* < .05), GSE41130, and GSE61853 than in the controls. In GSE18366 in particular, HIF‐1α expression was considerably higher in MDS patients than in the controls (*P* < .05) and statistically significant according to the receiver operating characteristic (ROC) analysis area under the curve (AUC) = 0.77 (*P* < .05). Compared with the controls, HIF‐1α expression was lower in GSE4619 (*P* < .05), GSE19429 (*P* < .001), GSE30195 (*P* < .05), GSE30201 (*P* < .01), GSE43399, GSE51757, GSE58831 (*P* < .01), GSE81173, GSE100340, and GSE15061 (*P* < .01).

**Table 3:** Basic information about the microarrays obtained from the GEO database and included in the research

ID	Public year	Country	Public journal	First author	Matched	Sample type	Cell type	Sample number			
								Control	N	Case	N
GSE2779	2005	UK	Blood	Sternberg A	Age‐matched	BM	MC	Healthy control	9	MDS	14
GSE4619	2006	UK	Blood	Pellagatti A	NR	BM	MC	Healthy control	11	MDS	55
GSE18366	2009	USA	Blood	Sridhar K	Age‐matched	BM	MC	Healthy control	6	MDS	35
GSE19429	2010	UK	Leukemia	Pellagatti A	NR	BM	MC	Healthy control	17	MDS	183
GSE30195	2011	USA	Nat Genet	Graubert TA	NR	BM	CD34 + cell	Healthy control	4	MDS	15
GSE30201	2011	USA	Blood	McGowan KA	Age‐matched	BM	MC	Healthy control	11	MDS	8
GSE25300	2012	Italy	Haematologica	Pigazzi M	Paired	BM	CD34 + cell	AML evolution	4	MDS	4
GSE41130	2012	Spain	Leukemia	del Rey M	Age‐matched	BM	MC	Healthy control	7	MDS	18
GSE43399	2013	Germany	Elife	Heinrichs S	NR	BM	MC	Healthy control	4	MDS	26
GSE51757	2013	China	Br J Haematol	Zhao X	Age‐matched	BM	MC	Healthy control	4	MDS	4
GSE58831	2015	UK	Nat Commun	Gerstung M	NR	BM	MC	Healthy control	17	MDS	159
GSE61853	2015	South Korea	PLoS One	Kim M	N	BM	MSCs	No lymphoma staging	7	MDS	7
GSE81173	2016	China	Sci Rep	Xu F	N	BM	MC	Healthy control	6	MDS	12
GSE100340	2017	China	Leukemia	Zhang T	NR	BM	MC	Healthy control	6	MDS	7

Abbreviations: AML, acute myeloid leukemia; BM, bone marrow; GEO, Gene Expression Omnibus; MC, mononuclear cell; MDS, myelodysplastic syndrome; MSCs, mesenchymal stromal cells; N, No; NR, not reported; PB, peripheral blood.

### 3.3 Meta‐analysis of HIF‐1α expression in MDS‐related microarrays and diagnostic test

**Figure 7:**
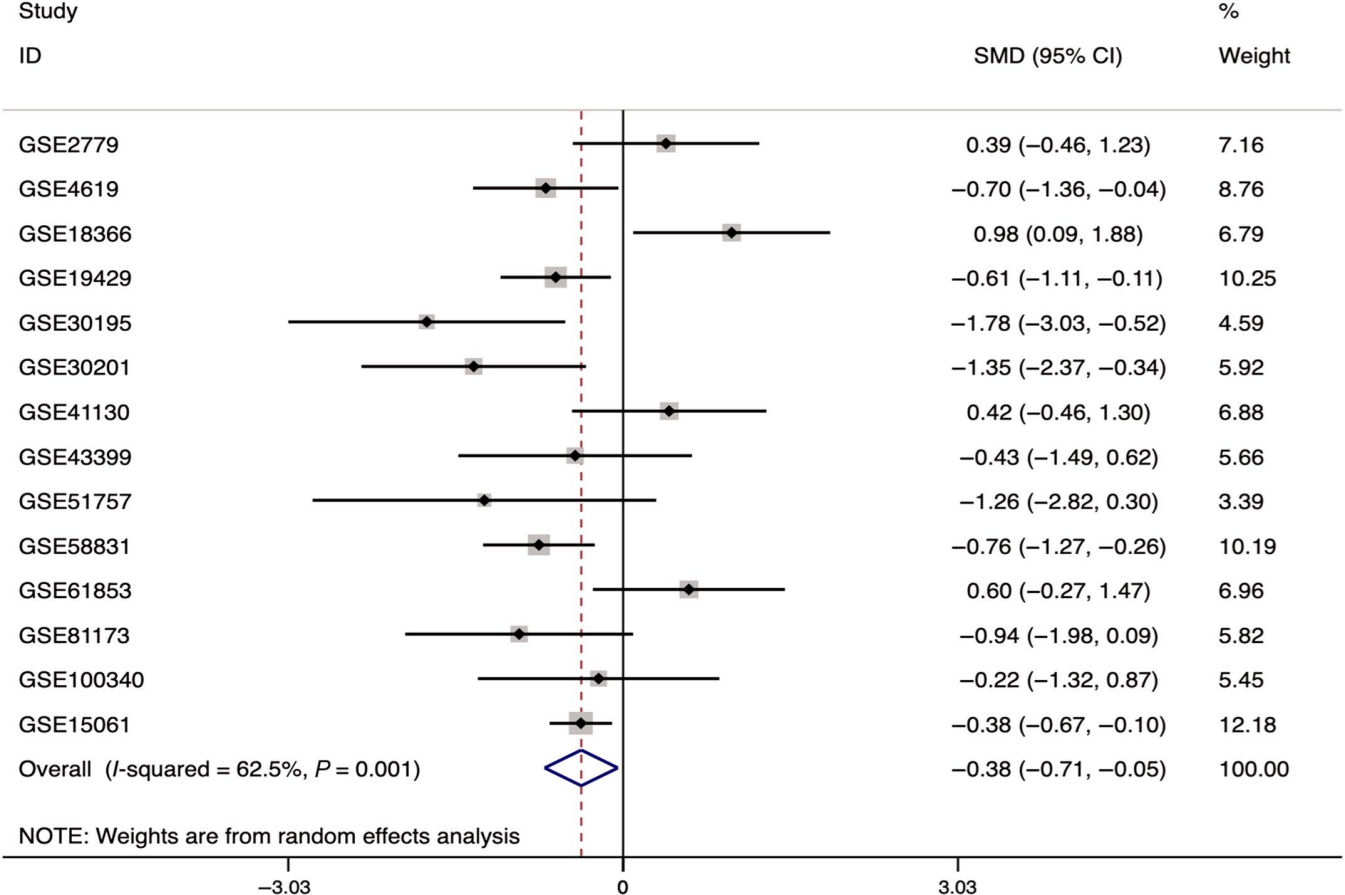
Forest plot of HIF‐1α expression level in 14 microarrays related to MDS and the control group. HIF‐1α, hypoxia‐inducible factor 1 alpha; MDS, myelodysplastic syndrome; SMD, standardized mean difference

**Figure 8:**
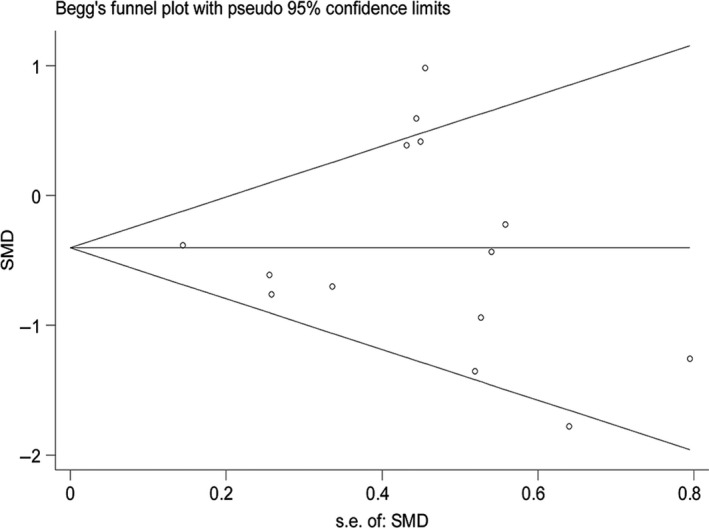
Funnel plot of HIF‐1α expression level in 14 microarrays related to MDS and the control group. HIF‐1α, hypoxia‐inducible factor 1 alpha; MDS, myelodysplastic syndrome; SMD, the standardized mean difference

**Figure 9:**
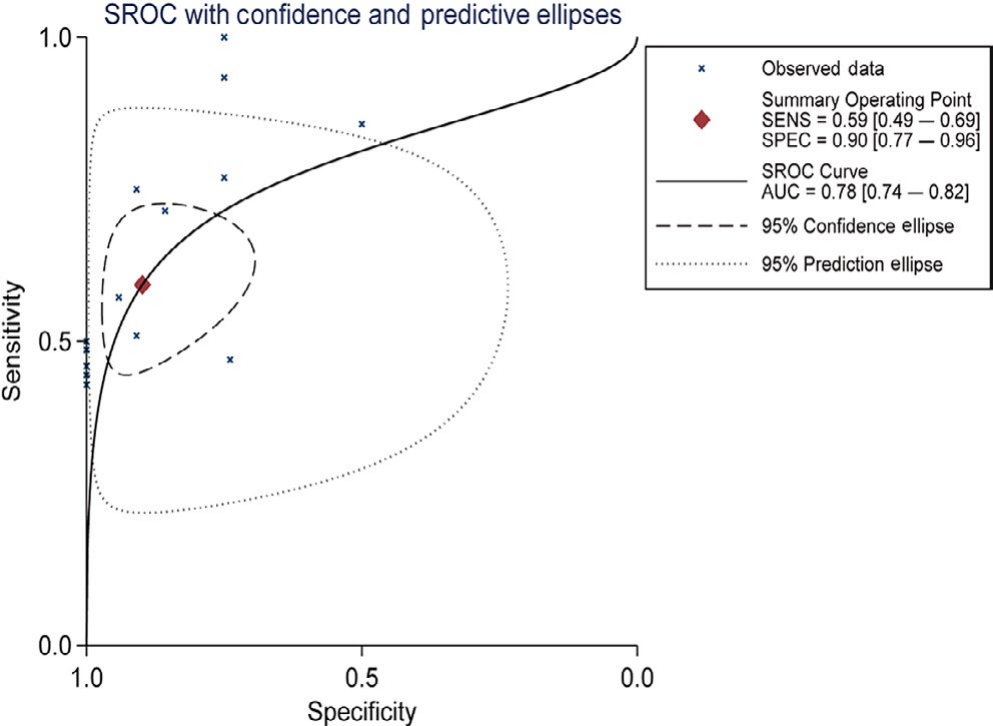
SROC curve of HIF‐1α in 14 microarrays related to MDS and the control group. AUC, area under the curve; HIF‐1α, hypoxia‐inducible factor 1 alpha; SENS, sensitivity; SPEC, specificity; SROC, summary receiver operating characteristic

Meta‐analysis using the standardized mean difference (SMD) was performed on HIF‐1α expression in the 14 microarrays, which included the MDS cohort as well as the controls. In these 14 microarrays, a heterogeneity test showed that *I* ^2^ = 62.5% and *P* = .001, so the random effects model was applied. Also, the meta‐analysis revealed that SMD = −0.38 (−0.71, −0.05), *z* = 2.24, and *P* = .025, with statistical significance in the differential expression. In the microarrays, the researchers observed a downregulated expression of HIF‐1α in the MDS cohort (Figures 7 and 8). In addition, meta‐analysis of the diagnostic test of the 14 microarrays revealed that the sensitivity of HIF‐1α was 0.59 (0.49‐0.69), the specificity was 0.90 (0.77‐0.96), and the AUC was 0.78 (0.74‐0.82; Figure 9).

### 3.4 HIF‐1α targeting miR‐93‐5p

**Figure 10:**
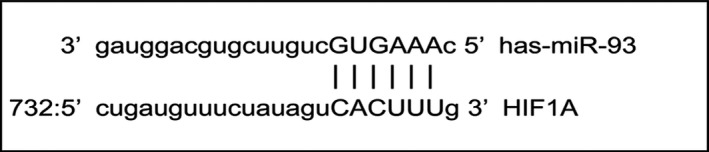
The site at which miR‐93‐5p binds to HIF‐1α. HIF‐1α, hypoxia‐inducible factor 1 alpha; miRNA, microRNA

**Figure 11:**
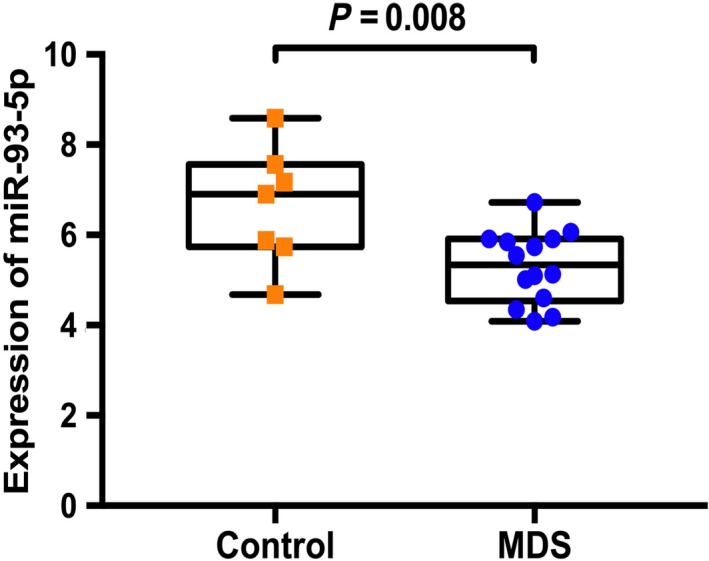
Expression of miR‐93‐5p in 14 MDS patients and 7 controls from a GSE76775 microarray. MDS, myelodysplastic syndrome; miRNA, microRNA

The researchers used the four databases on miRWalk2.0 (miRWalk, miRanda, RNA22, and Targetscan) to predict the target miRNAs of HIF‐1α. It was discovered that miR‐93‐5p had target sites for HIF‐1α (Figure 10). Also, miR‐93‐5p was differentially expressed miRNA in the MDS cohort and the controls. In GSE76775, miR‐93‐5p exhibited the lowest expression in the MDS cohort (Figure 11).

### 3.5 The dual luciferase experiment: miR‐93‐5p could directly target HIF‐1α

**Figure 12:**
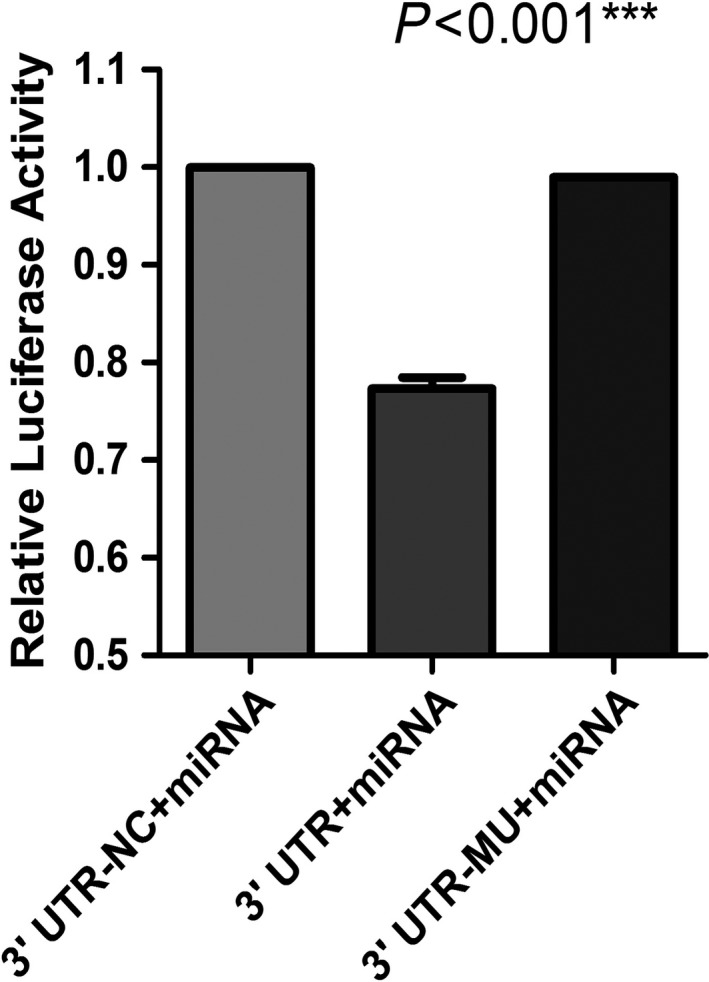
Results from the dual luciferase reporter assay system showing that miR‐93‐5p and HIF‐1α have complementary sequences. HIF‐1α, hypoxia‐inducible factor 1 alpha; miRNA, microRNA. ***P < .001

With the assistance of bioinformatics techniques and the differentially expressed miRNA in GSE76775, the researchers discovered that miR‐93‐5p was the target gene of HIF‐1α. Complementarity was also found between HIF‐1α and miR‐93‐5p, which indicated that miR‐93‐5p and HIF‐1α were likely to combine in cells to fulfill biological roles and influence cell functions. To molecularly explore the target relationship and determine whether miR‐93‐5p could target HIF‐1α, a dual luciferase experiment was carried out on 293T‐cell lines. Blank control, wild‐type, and mutant‐type HIF‐1α plasmids and overexpressed miR‐93‐5p plasmids were transfected into 293T cells. The experiment revealed that overexpressed miR‐93‐5p could lead to a remarkable reduction in the luciferase activity in the wild‐type group (*P* < .01) compared with the blank control, while overexpressed miR‐93‐5p resulted in obviously higher luciferase activity in the mutant‐type group than in the wild‐type group (*P* < .001; Figure 12), which confirmed that miR‐93‐5p had target sites for HIF‐1α.

## 4 DISCUSSION

HIF‐1 has been extensively detected in the cells of mammals. In hypoxic conditions, HIF‐1 was involved in transcriptional induction of various genes that participated in physiological reactions like angiogenesis, glucose metabolism, cell proliferation, cell survival, and regulation.^50^, ^51^, ^52^ HIF‐1α, a subunit of HIF‐1, possesses the ability to determine HIF‐1 activity and plays a leading role in hypoxia response. Tumor cells’ accommodation for hypoxic conditions is of paramount importance in tumor progression.^53^

The previous experiments in this research demonstrated that the mRNA of HIF‐1α displayed considerably higher expression in MDS patients (*P* < .001). This high expression was related to factors associated with poor prognosis (*P* < .05), including WHO classification, chromosomal abnormalities, and IPSS scores. Using IHC, this research explored the protein level and expression significance of HIF‐1α and PDGF‐B in MDS and AML patients. The experiment showed that, compared with the 33 controls, the protein level of HIF‐1α was upregulated in the 82 MDS cases (*P* < .05). Regarding the clinical parameters, the researchers observed that the positive expression rate of HIF‐1α was higher in the RAEB1/RAEB2 cohort than in the RA/RARS/ RCMD/5q cohort, the rate of HIF‐1α (+) was higher in the initial cell ≥5% cohort than in the initial cell <5% cohort, and the mean value of lactate dehydrogenase was greater in the HIF‐1α (+) cohort than in the HIF‐1α (−) cohort. Based on these results, we conclude that the MDS patients with positive HIF‐1α expression tended to have a poor prognosis, since RAEB1/RAEB2 was likely to convert to AML, the percentage of initial cells (>5%) was associated with poor prognosis, and the increased level of lactate dehydrogenase was correlated with unsatisfactory prognosis for hematologic malignancies. In addition, for patients with positive HIF‐1α expression, the mean survival time was shorter in cohorts with factors related to poor prognosis (including the RAEB1/RAEB2, unsatisfactory prognosis, high risk, and initial cell ≥5% cohorts; *P* < .05). This indicates that HIF‐1α was upregulated in MDS patients and connected with factors related to poor prognosis, such as conversion to AML, increasing percentage of initial cells, increasing lactate dehydrogenase level, and short survival time.

Du et al^54^ found that HIF‐1α expression was notably higher in the 48 examined myeloid tissue samples from MDS patients than in the 20 samples from the controls. Furthermore, HIF‐1α was more highly expressed in the RAEB1/RAEB2 cohort than in the RA/RARS/RCMD/5q‐ cohort, in the IPSS ≥1.5 cohort than in the IPSS <1.5 cohort, and in the chromosome abnormality cohort than in the normal controls. These results indicate that high HIF‐1α expression is associated with unsatisfactory prognosis, consistent with our research.

A large number of studies have demonstrated that HIF‐1α plays a role in the carcinogenesis of solid tumors. HIF‐1α was highly expressed in the majority of tumors examined in these studies, indicating poor prognosis. Wang et al^55^ used a quantitative real‐time polymerase chain reaction and IHC to investigate HIF‐1α expression in pancreatic carcinoma. They found that HIF‐1α expression was increased in most patients, with weakly positive expression in most cancer tissues and strongly positive expression in the adjacent tissues. Clinicopathologic analysis revealed that the strongly expressed HIF‐1α in the cancer group was associated with the depth of tumor invasion, pathologic stage of cancer, advanced stage according to the American Joint Committee on Cancer classification, and shorter overall survival time. Therefore, it was regarded as a factor associated with unsatisfactory prognosis.

For comprehensive analysis of HIF‐1α expression in MDS patients, a total of 14 microarrays from the GEO and ArrayExpress databases were included in this research. In GSE18366, HIF‐1α was obviously expressed more highly in the MDS cohort than in the controls, and a diagnostic test of ROC confirmed that the expressed HIF‐1α could help to diagnose MDS with statistical significance. These results were consistent with the results of our IHC experiment. Data concerning the expression of HIF‐1α were processed by a meta‐analysis that combined a continuous variable and SMD. The analysis revealed that SMD = −0.38 (−0.71, −0.05), *z* = 2.24, and *P* = .025, with statistical significance in the differential expression. According to the microarrays, HIF‐1α expression was downregulated in MDS. In addition, the meta‐analysis of SROC showed that the AUC was 0.78 (0.74‐0.82), demonstrating the moderate value of HIF‐1α for diagnosing MDS. In the four microarrays related to AML and MDS, greater heterogeneity was detected in general HIF‐1α expression, with the diamond intersecting with the vertical line (line of no effect; Figure 7). The AUC of HIF‐1α was 0.78 (0.74‐0.82), indicating that HIF‐1α had moderate prognostic capability for AML.

The meta‐analysis combining continuous variables and SMD demonstrated that downregulated expression of HIF‐1α occurs in MDS patients. This could be explained by the different test platforms and methods used to deal with the microarrays. These circumstances may have led to the inconsistency between the results of meta‐analysis and IHC.

Recent research has determined that oncomiR (miR‐93‐5p) plays an essential role in the onset and development of various tumors. Jiang et al^56^ discovered that, in hypoxic conditions, NF‐κB expression increased in hepatoma cells, and the Bp50 and p65 NF‐κ subunits bound to the HIF‐1α promoter, thereby increasing transcription. In addition, miR‐93‐5p was the downstream target of NF‐κ c‐Rel subunits and was able to reduce the mRNA and protein level of HIF‐1α. Also, miR‐93‐5p was related to gastric cancer. Li et al^57^ found that the expression of miR‐93‐5p was increased in gastric cancer tissues, and overexpression of miR‐93‐5p promoted the proliferation, metastasis, invasion, and chemical resistance of tumor cells. The findings of Shyamasundar et al^58^ demonstrated that overexpressed mature miR‐93‐5p in MDA‐MB‐231 breast cancer cells can suppress cell migration, invasion, and adhesion. Suppression of miR‐93 caused contrary results. Wang et al^59^ found that, in patients with hepatocellular carcinoma, miR‐93‐5p can directly target 3'‐UTR in the mRNA of PPARGC1A (also known as PGC‐1a), inhibiting the expression of PPARGC1A, which functions as a coactivator of transcription and a metabolic modulator. Yang et al^60^ revealed the relationships between miR‐93‐5p expression and the overall survival rate of patients with non‐small cell lung carcinoma (NSCLC). In NSCLC patients, miR‐93‐5p expression was upregulated and exerted a carcinogenic role by inhibiting PTEN and RB1. This suggests that miR‐93‐5p might act as a prognostic indicator and therapeutic target.

In conclusion, this research found that HIF‐1α expression was upregulated in the myeloid tissues of MDS patients. Such upregulation was associated with the factors related to unsatisfactory prognosis. In addition, HIF‐1α correlated with angiogenesis. The researchers also confirmed that HIF‐1α can bind to miR‐93‐5p by targeting. Based on these results, it is presumed that, in MDS patients, HIF‐1α will bind to miR‐93‐5p by targeting and participate in angiogenesis in the bone marrow, thereby fulfilling its biological functions in the initiation and progression of MDS. More research is required to confirm these results.

## CONFLICT OF INTEREST

None declared.
